# Developing the health, safety and environment excellence instrument

**DOI:** 10.1186/1735-2746-10-7

**Published:** 2013-01-07

**Authors:** Iraj Mohammadfam, Gebraeil Nasl Saraji, Ali Kianfar, Shahram Mahmoudi

**Affiliations:** 1 Department of Occupational Health, Hamadan University of Medical Sciences, Hamadan, Iran; 2 Department of Occupational Health, Tehran University of Medical Sciences, Tehran, Iran; 3 Department of HSE, Mapna Group, Tehran, Iran

**Keywords:** Assessment, Health, Safety, Environment, Management systems

## Abstract

Quality and efficiency are important issues in management systems. To increase quality, to reach best results, to move towards the continuous improvement of system and also to make the internal and external customers satisfied, it is necessary to consider the system performance measurement. In this study the Health, Safety and Environment Excellence Instrument was represented as a performance measurement tool for a wide range of health, safety and environment management systems. In this article the development of the instrument overall structure, its parts, and its test results in three organizations are presented. According to the results, the scores ranking was the managership organization, the manufacturing company and the powerhouse construction project, respectively. The results of the instrument test in three organizations show that, on the whole, the instrument has the ability to measure the performance of health, safety and environment management systems in a wide range of organizations.

## Introduction

Human resources are the key factor of sustainable development in modern management [1]. Different approaches have been considered for achieving industrial world and sustainable development, but regardless of human resources, no one would be successful and even the best designed systems will fall down [2]. The utilization of modern technology has developed industries that are more complex in the last decade [3]. Therefore, preservation of human health and safety is the modern management approach and one of the essential elements in organizations policy [2].

If modern organizations want to survive in the dynamic and competitive environment of today they have to consider safety and welfare of their employees as well as protection of environment in addition to a desire to promote customer satisfaction [4]. In addition, safety, health, and environmental factors have been emphasized in national and international regulation and litigation (including Iran's national regulations) [5]. Furthermore, safety, health, and environmental factors are delicate subjects for customers, employees and other stakeholders [6]. Therefore, organizations that choose to pay closer attention to these basics would experience even more success on the way to continuous improvement. With the aim of meet these needs, Management Systems standards such as ISO 9000, ISO 14000, and Occupational Health and Safety Advisory Services (OHSAS) 18000 have developed.

The main objective in implementation of the requirements for such management systems is to ensure that safety, health and environmental issues are being addressed in organizations’ strategies [7]. In each of the systems mentioned, only one aspect of the organizations is considered (e.g., quality in ISO 9000, environmental protection in ISO 14000 or safety and health in OHSAS 18000). On the one hand the role of such management systems in administrating activities due to organizations’ policies and strategies is unquestionable, but on the other hand multiplicity of systems can cause difficulty and confusion of organizations, duplication, resource waste, policies and goals conflicts [8,9]. Thus, the integration of management systems is obligatory [10].

Considering the trend of significant investments in establishing different types of integrated management systems like Health, Safety and Environment Management System (HSE-MS) in Iran, it is therefore crucial to provide an answer to the question whether the implementation of integrated management systems increase productivity? Consequently, evaluating the effects of deployment of these systems would be required [11]. Another factor that makes these evaluations necessary are bound to financial constraints that organizations often face when they want to establish an effective and efficient integrated management system.

Despite the attention given to HSE management systems, there has been relatively little attention given to the measurement of HSE-MS performance. The few past studies have considered just one or two parts of HSE management systems. Some of these studies are as follows:

1. Carder and Ragan over a 10-year period (1991–2001), through working in a chemical company with about 6000 employees and over 50 plants studied the use of employee surveys to measure safety and as a diagnostic tool for improvement efforts. The survey measures important components of the safety management system [12].

2. In 1998, The Michigan Occupational Health and Safety Management System Assessment Instrument (MAI) renamed in 2002 to Universal Assessment Instrument (UAI) [13,14], which measures the performance of OHS management systems and which was developed by researchers from the University of Michigan, reported numerous cases of large companies that have improved their OHS results through the use of this tool.

3. Cadieux *et al.,* (2005) introduced an instrument designed to conduct an OHS self-diagnosis. Their tool was a questionnaire using a 10 anchor-point. The study was done in three companies [4].

4. Mohammad Fam *et al.,* (2008) introduced an instrument designed to evaluate the effects of Health, Safety, and Environment Management Systems on organizations based on Balanced Score Card (BSC).

The present article describes an instrument designed to measure HSE-MS performance based on three input models. The main objectives of the instrument is:1) making it possible for the organizations to identify their position comparing with other organizations and ranking them independent of their business nature, 2) providing a self-assessment tool, and 3) not being just an audit tool but also a managerial tool that identifies system problems and can help management to provide corrective actions.

## Materials and methods

### Input model selection

Nine performance measurement models of Analytic Hierarchy Process (AHP), Balanced Score Card (BSC), Goal programming (GP), Data Envelopment Analysis (DEA), Re-Engineering (REE), European Foundation for Quality Management (EFQM), Malcolm Baldrige National Quality Award (MBNQA), International Project Management Award (IPMA), and The Safety Culture Improvement Matrix (SCIM) were reviewed in an effort to define the Health, Safety and Environment Excellence Instrument (HSEEI).

Three of these models were selected as instrument input models. These models are:1) EFQM, 2) IPMA, and 3) SCIM because they are widely applicable, easy to administer and their outcome determinations is easy to interpret. The instrument first included 11 parts.

### Developing instrument structure

The instrument in the form of a specific questionnaire was sent for 75 HSE specialists and experts. In this questionnaire, experts were asked to: 1) evaluate instrument parts in their point of view, 2) prioritize them, and 3) use paired comparisons method to determine score and the proportional coefficient of each part comparing to other parts. The instrument structure and the scores of its parts were determined by reviewing the completed collected questionnaires.

Sub-parts and guidance points were determined through the experts' opinions and using EFQM, IPMA, and SCIM models.

The structures of three input models were reviewed and the HSE experts’ opinions were taken in order to determine how the instrument can be used to measure the performance of Health, safety and environment management system.

### Instrument finalizing

In order to calibrate the proposed instrument, it was first tested in a small organization and necessary modifications based on test findings were applied. Then, the proposed instrument was tested in three different sub sectors of a great industrial company in Iran. The goals of the tests were: 1) to evaluate the ability of the HSEEI in organizations with different types of business natures, 2) to evaluate and further develop of the HSEEI. For this purpose, the first organization was a powerhouse construction project, the second one was a manufacturing company and the third was a managership organization.

## Results

The HSEEI was based on the principles of three input models (EFQM, IPMA and SCIM) and expert opinions. The HSEEI is basedon 9 parts to evaluate organizations and help them in the way to achieve HSE excellence. The HSEEI is not just an audit tool but also a managerial tool that includes a variety of techniques and improvement frameworks in proportion to the level of excellence in the organization to identify system problems and help management to provide corrective actions. Organizations that are in the beginning of the way to HSE excellence can utilize the HSEEI for self-assessment and measurement of their level in HSE excellence.

**Figure 1:**
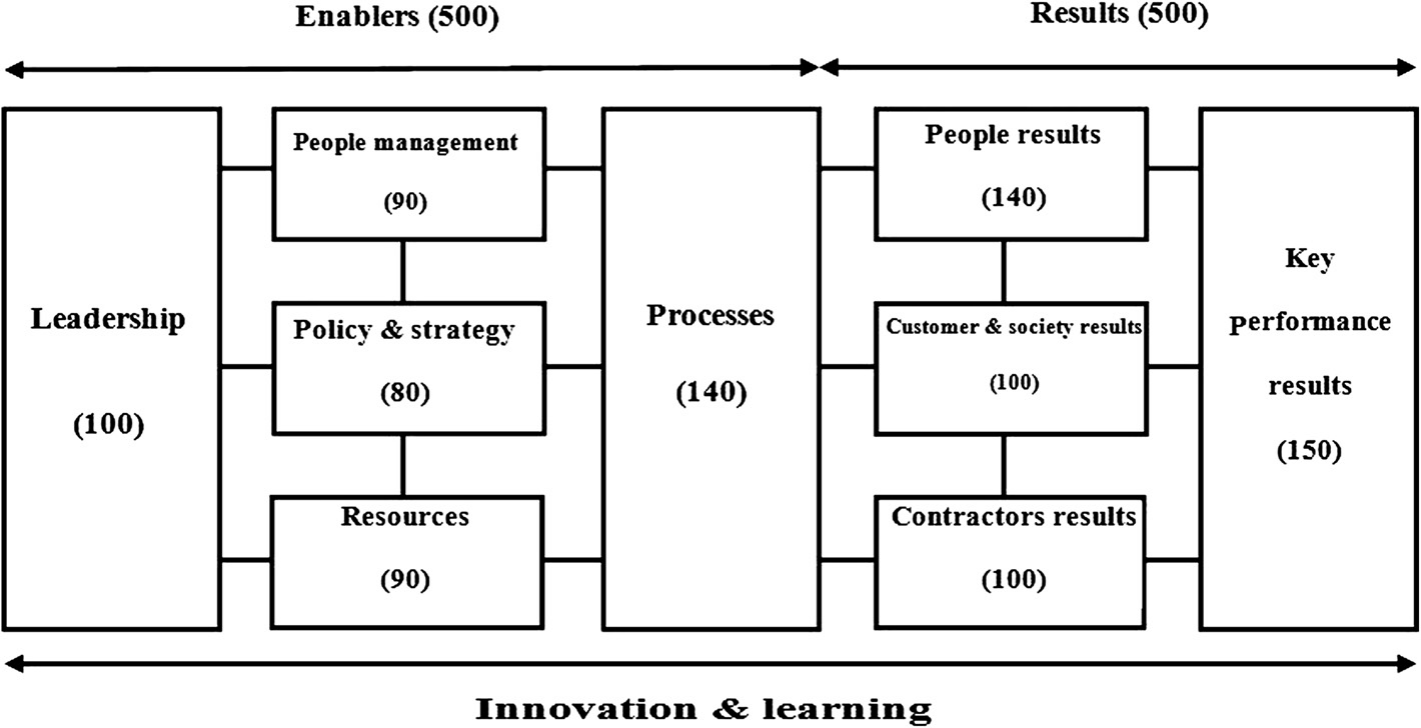
The HSEEI scheme.

The main part of HSEEI is shown in Figure 1. Each part of HSEEI is divided into a number of sub-parts and each sub-parts into a number of guidance points. The HSEEI includes the concept of distinguishing between “enablers” and “results”, and in its initial form developed by HSE specialists and experts, contains 9 parts, 33 sub-parts, and 370 guidance points. After the test in a small organization was done (for calibration), changes have been made to the context of the parts, sub-parts and guidance points. The HSEEI in its final form contains 9 parts, 38 sub-parts, and 410 guidance points. Addressing all guidance points is not required by HSEEI and organizations should consider them to their specifications.

The structure varies somewhat with enablers and results. Results are divided into two sub-parts as follows:

A. Perception measures: These measures represent imagination and inference of employees, customers, contractors and the society about the organization.

B. Performance indicators: These indicators are internal criteria that organization uses to view, understand and predict the imagination and inference of employees, customers, contractors and society and improving its performance in respect to them.

Part 9 is divided into two sub-parts as follows:

A. Key performance outcomes: These indices are key results defined by the organization and have been agreed in its policy and strategy.

B. Key performance indicators: These indicators are operational measures that organization use them to view, understand, predict and improve key performance outcomes.

**Figure 2:**
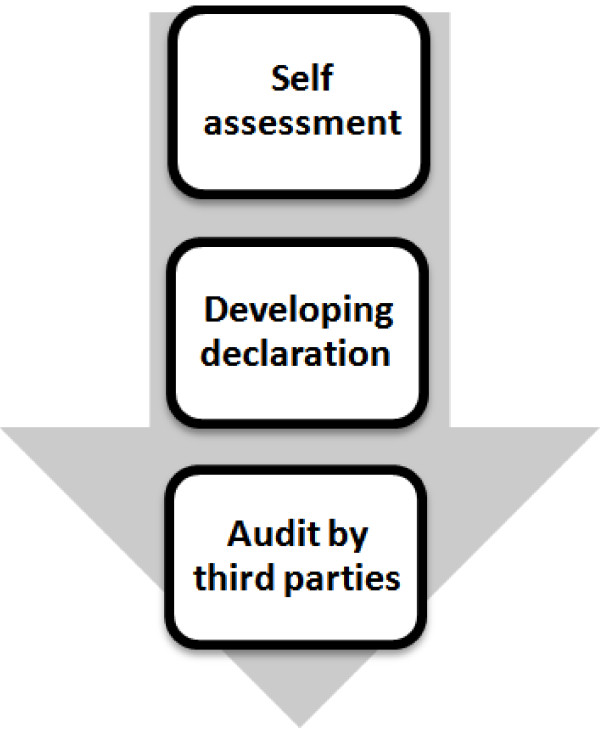
The structure of the measurement in HSEEI.

Of course, for each sub-part in results some guidance points are defined. Table 1 lists the number of sub-parts and guidance points used in HSEEI. The HSE experts' opinions and the structures of three input models (EFQM, IPMA and SCIM) were reviewed to determine how HSEEI measures the implementation of the HSE-MS established in an organization. Figure 2 outlines the structure of the measurement. In this study, the RADAR logic (Results, method and approach, the current spread or building, evaluation and revision) that has been used in EFQM and IPMA was used in auditing by third party. Checklists were developed for HSEEI third party audits including questions that evaluator should give them a score between 0 to 100 based on the contents of the part or sub-part that is under review or even evaluator can consider guidance points but it is not mandatory. The HSEEI was tested in three different sub sectors of a great industrial company in Iran. Table 2 illustrates results of the third party audit.

**Table 1:** Number of sub-parts and guidance points in HSEEI

**Part**	**Number of sub-parts**	**Number of guidance points**
Leadership	8	93
Policy and strategy	5	43
People management	6	60
Resources	7	54
Processes	4	29
Customer and society results	2	43
People results	2	36
Contractor results	2	33
Key performance results	2	19
**Total**	**38**	**410**

**Table 2:** Results of the third party audit

**Part**	**Org. 1**	**Org. 2**	**Orgcpa 3**	**Maximum score in HSEEI**
Leadership	25.7	31.25	34.5	100
Policy & strategy	26.52	39.33	41.33	80
People management	21.8	32.77	26.6	90
Resources	21.34	18.33	24.34	90
Processes	25.42	35.41	41.17	140
Total score in enablers	120.86	157.09	167.94	500
Customer & society results	31.25	28.75	33.8	100
People results	21.87	28.12	31.2	150
Contractor results	28.12	31.25	29.72	100
Key performance results	31.25	34.37	41.27	150
Total score in results	112.49	122.49	135.99	500
Total score (enablers + results)	233.35	279.58	303.93	1000

## Discussion

The most important strengths and weaknesses of the HSEEI revealed by its test in three different types of organizations. Contrary to Carder and Ragan (1991–2001) method that can be used only in chemical process industries [12] and Mohammad Fam *et al*., (2008) study that was not tested in any organization. Because the HSEEI independent structure (being proved by test findings in three different sub sectors of a great industrial company), it can measure the performance and efficiency of a wide range of HSE management systems [15]. The proposed instrument can be used to measure the performance and efficiency of health, safety and environment management systems not just safety like Carder and Ragan (2003) approach or safety and health like UAI (Redinger and Levine, 1998 and 2002) and Cadieux *et al.,* (2005) and it is not just based on a questionnaire with fixed questions like Carder and Ragan (2003) approach and Cadieux *et al*., (2005) study [4,12-14].

Widespread application of three input models especially the EFQM that is also extensively used as the national productivity award in Iran can be called as strength in HSEEI, because of widespread awareness that exists in organizations particularly about EFQM. This awareness results in shorter time lost by organizations to gain knowledge, skill, and understanding in HSEEI through training. Using the HSEEI in three different organizational structures revealed that the methods used were effective in collecting the necessary information to further understanding the complex nature of management system measurement. The HSEEI provides a valid framework for organizations to follow in their development of a HSE-MS performance measurement system. The key advantages of HSEEI for organizations are as follows:

1. Determining the current level of organization and Possibility of quantitative analysis.

2. Organizations could identify their strengths and weaknesses and classify the improvement programs as well as administering the trade and pace in the way to HSE excellence.

3. The HSEEI helps organizations to compare themselves with industry leaders in each HSEEI part.

4. The HSEEI helps organizations to balance the expectations and requirements of stakeholders, i.e. customers, general public, employees and local authorities.

5. The HSEEI can be implemented in a wide range of systems and organizations because of its process approach

6. In the long run, the HSEEI provides a systematic point of view on specifications of an excellent organization in HSE management system.

7. The HSEEI, because of its concept of enablers, provides an opportunity for organizations to pass from the reactive actions (i.e., developing preventive actions after accidents occur) to proactive actions (i.e., developing preventive actions before accidents occur).

When utilizing HSEEI to evaluate a HSE management system, the following notes should be considered primarily:

Organization should provide effective training for all employees (including employees who are implementing self assessment) involved in HSEEI activities.

Organization should assign appropriate resources for utilizing the HSEEI.

Lack of practical experience of the HSEEI in organizations possibly will cause it to proceed slowly.

To compare the performance of HSE-MS in different organizations and in order to encourage them to use this instrument, it is suggested to found and develop a HSE excellence award.

## Conclusion

By testing the HSEEI in three organizations with different types of business natures revealed that the instrument was effective in collecting the necessary information and in performance measurement of different management systems with complex natures. The HSEEI provides a valid framework for organizations to follow their trend of Health, Safety and environment management system development.

## Competing interests

The authors declare that they have no competing interests.

## Authors’ contributions

IM and AK were involved in wrote of the first draft of the manuscript and discussion of the results and edited the manuscript. GNS was involved in the discussion of the results and read the manuscript. All authors read and approved the final manuscript.
